# Frequent discordance in PD-1 and PD-L1 expression between primary breast tumors and their matched distant metastases

**DOI:** 10.1007/s10585-018-9950-6

**Published:** 2018-12-13

**Authors:** Quirine F. Manson, Willemijne A. M. E. Schrijver, Natalie D. ter Hoeve, Cathy B. Moelans, Paul J. van Diest

**Affiliations:** Department of Pathology, University Medical Center Utrecht, PO Box 85500, 3508 GA Utrecht, The Netherlands

**Keywords:** Programmed death-1, Programmed death-ligand 1, Breast cancer, Metastasis, Prognosis

## Abstract

Programmed death-1 (PD-1) is an immune checkpoint that is able to inhibit the immune system by binding to its ligand programmed death-ligand 1 (PD-L1). In many cancer types, among which breast cancer, prognostic and/or predictive values have been suggested for both PD-1 and PD-L1. Previous research has demonstrated discrepancies in PD-L1 expression between primary breast tumors and distant metastases, however data so far have been scarce. We therefore evaluated immunohistochemical expression levels of PD-1 and PD-L1 in primary breast tumors and their paired distant metastases, and evaluated prognostic values. Tissue microarrays from formalin-fixed paraffin-embedded resection specimens of primary breast cancers and their matched distant metastases were immunohistochemically stained for PD-1 and PD-L1. PD-1 was available in both primary tumor and metastasis in 82 patients, and PD-L1 in 49 patients. PD-1 was discrepant between primary tumor and metastasis in half of the patients (50%), PD-L1 on tumor cells was discrepant in 28.5%, and PD-L1 on immune cells in 40.8% of the patients. In primary tumors there was a correlation between PD-1 positivity and a higher tumor grade, and between immune PD-L1 and ER negativity. In survival analyses, a significantly better overall survival was observed for patients with PD-L1 negative primary breast tumors that developed PD-L1 positive distant metastases (HR 3.013, CI 1.201–7.561, p = 0.019). To conclude, PD-1 and tumor and immune PD-L1 seem to be discordantly expressed between primary tumors and their matched distant metastases in about one-third to a half of the breast cancer patients. Further, gained expression of PD-L1 in metastases seems to indicate better survival. This illustrates the need of reassessing PD-1 and PD-L1 expression on biopsies of distant metastases to optimize the usefulness of these biomarkers.

## Introduction

Programmed death-1 (PD-1) is an immune checkpoint that is able to inhibit the immune system by binding to its ligand programmed death-ligand 1 (PD-L1), and prohibits T cell function in this way. In literature it has been described that in different cancer types [1], especially immunogenic tumors, including non-small cell lung cancer [2], malignant melanoma [3], and ovarian cancer [4], PD-1 can be upregulated on tumor infiltrating lymphocytes (TILs) and PD-L1 on tumor and immune cells. These markers could have potential prognostic and/or predictive values [5]. Although breast cancer is not considered very immunogenic, expression of PD-1 and PD-L1 has been described and prognostic and predictive values have been suggested, albeit with varying results [6–17].

Most studies focused on the expression of PD-1 and/or PD-L1 in primary breast tumors. However, through selective metastases of subclones, or tumor progression, expression levels and prognostic values in metastases may differ from the primary tumors. For example, previous research has described receptor conversion for ER, PR, HER2 and several molecular imaging targets in a significant proportion of breast cancer metastases [18, 19]. Additionally, it has been observed that conversion from ER/PR-positive primary breast cancers to ER/PR-negative metastases is associated with negative prognosis [20].

Since biomarker expression status of distant metastases, rather than the primary tumor will dictate response to targeted therapy, it is important to know whether conversion from the primary tumor to distant metastases also occurs for PD-1 and PD-L1. Two previous studies described high concordance of PD-L1 expression in primary breast tumors and their matched lymph node metastases, and lower PD-L1 expression levels in non-matched distant metastases, respectively [21, 22]. One study comparing PD-L1 expression between metastatic and non-metastatic primary breast cancers described one case where PD-L1 expression in primary tumor and metastasis was concordant, while in the second case expression was discordant [23]. As far as we know, these previous studies have only evaluated PD-L1 expression, while PD-1 expression may be of importance as well, certainly since brain metastases, compared to their primary breast tumor, are thought to have a lower amount of TILs [24].

Given the limited data on PD-1 expression and the anecdotal data on PD-L1 expression in primary breast cancers compared to their matched distant metastases to date, we compared immunohistochemical expression levels of PD-1 and PD-L1 in primary tumors and their matched distant metastases in a large group of metastatic breast cancer patients, and evaluated prognostic values.

## Materials and methods

### Patients and samples

For previous research, tissue microarrays (TMAs) with 106 primary female breast cancers and their matched distant metastases from various distant anatomical sites (bone, brain, gastrointestinal, gynecological (uterus/ovary), liver, lung/pleural and skin/subcutis) had been assembled [25, 26]. All metastases were metachronous metastases. Clinicopathological data (age, tumor size, histology, grade (according to the modified Bloom and Richardson score) [27], lymph node status, and surrogate molecular subtype, based on hormone receptor status—the ER/PR we used was, according to the Dutch guidelines with a cut-off of 10%, and HER2 was scored according to the ASCO/CAP guidelines) were anonymously extracted from digital reports/revised by a pathologist specialized in breast cancer (PvD). Follow-up data were anonymously obtained through the Comprehensive Cancer Center of The Netherlands (IKNL). Use of anonymous or coded left over material for scientific purposes is part of the standard treatment agreement with patients and therefore ethics approval and informed consent procedure was not required according to Dutch legislation [28].

### Immunohistochemistry

The TMAs contained three cores of 0.6 mm from representative areas in formalin-fixed paraffin-embedded (FFPE) tissue blocks of both primary tumor and metastasis. 4 µm thick tissue sections were cut and immunohistochemically stained with the Ventana autostainer (Roche, Tuscon, USA). For PD-1, we used a mouse anti-PD-1 monoclonal antibody (ab52587 (NAT105, dilution 1:50, Abcam, Cambridge, UK)) and for PD-L1, a rabbit anti-PD-L1 monoclonal antibody (741–4905 (clone sp263, dilution Ventana ready to use; Ventana Medical Systems, Tuscon, Arizona, USA)). ER (Ventana 1:100), PR (Dako 1:100) and HER2 (Neomarkers 1:100) were also stained with the Ventana autostainer.

### Assessment of PD-1 and PD-L1

The immunohistochemically stained slides were scored by consensus of two experienced observers (WS or QM and PvD), in random order and blinded to other data. PD-1 was scored positive in case of any membranous staining of immune cells. For PD-L1 we scored the positivity on both tumor cells and immune cells. Concerning PD-L1 on tumor cells, we scored the percentage of positive membranous staining. To create balanced groups for analyses, all cases with scores above 0 were considered to be positive. Immune PD-L1 was considered to be positive in case of any membranous staining of immune cells. Additionally, we verified the use of TMAs by comparing the PD-L1 scores of ten patients with PD-L1 scores on whole slides. In five cases, we observed complete agreement, and for the other five we only observed minimal differences.

### Statistics

Statistical analyses were performed with IBM Statistics 25. To study differences in hormone receptor expression and expression of PD-1 and PD-L1 between primary tumors and (matched) metastases, McNemar and Pearson Chi square tests were used. To compare expression of PD-1 and PD-L1 in metastases and their anatomical sites, we used Fisher’s exact test. For survival analysis, Kaplan–Meier curves were plotted and compared by log-rank test. Multivariate survival analysis was performed with Cox regression analyses. For all statistical tests, the significance level was set at p value < 0.05.

## Results

### Patient characteristics

Of the 106 primary tumors, PD-1 scores were obtained from 105 tumors and PD-L1 scores of 75 tumors. Regarding the 106 metastases, PD-1 scores were acquired of 83 tumors and PD-L1 scores of 67 tumors. Matched PD-1 scores were available for 82 tumors, while matched PD-L1 scores were available in 49 cases. Baseline clinicopathologic characteristics are shown in Table 1.

**Table 1:** Basic clinicopathologic data for metastatic primary breast cancers

Characteristics	Primary breast tumor
Age	
Mean [years (SD)]	52.6 (11.7)
Range	27–85
Tumor size	
Mean [cm (SD)]	3.3 (2.6)
Range	0.5–16.0
Histology^a^ [no. (%)]	
Ductal	90 (88.2)
Lobular	12 (11.8)
Grade [no. (%)]	
1	3 (3.0)
2	36 (35.6)
3	62 (61.4)
Lymph node status [no. (%)]	
Negative	45 (44.6)
Positive	56 (55.4)
Molecular subtype [no. (%)]	
Luminal	69 (65.7)
HER2 driven	12 (11.4)
Triple negative	24 (22.9)

^a^For the ductal type, also apocrine and metaplastic tumors were included

Mean age at surgery of the primary tumor was 53 years. The primary tumors had a mean size of 3.3 cm, most were ductal (88%), were grade 2 or 3, and two-third of the samples was of the luminal subtype (66%). More than half of the patients (55%) had positive lymph nodes at diagnosis of the primary tumor.

Table 2 demonstrates the frequency of ER, PR, and HER2 positivity in primary tumors and metastases. No significant differences were observed between the primary tumors and metastases concerning ER and HER2, but PR was significantly less often expressed in metastases compared to primary tumors. Concordances between primary tumors and their matched metastases were for ER 94%, for PR 78%, and for HER2 95% [Table 3].

**Table 2:** Receptor status in primary breast tumors and non-matched distant metastases

Characteristics	Primary tumor	Metastasis	p-value*
ER [no. (%)]			
Negative	44 (40.0)	39 (47.0)	0.332
Positive	66 (60.0)	44 (53.0)	
PR [no. (%)]			
Negative	52 (47.7)	52 (63.4)	**0.031**
Positive	57 (52.3)	30 (36.6)	
HER2 [no. (%)]			
Negative	88 (82.2)	66 (79.5)	0.635
Positive	19 (17.8)	17 (20.5)	

Bold indicates *p* < 0.05

*Pearson Chi square test

**Table 3:** Receptor expression in primary breast tumors compared to their matched distant metastases

Distant metastases									
Primary tumor	ER			PR			HER2		
	Negative	Positive	p-value*	Negative	Positive	p-value*	Negative	Positive	p-value*
Negative	34 (42.0%)	1 (1.2%)	0.375	38 (47.5%)	5 (6.3%)	0.096	63 (78.8%)	3 (3.8%)	0.625
Positive	4 (4.9%)	42 (51.9%)		13 (16.3%)	24 (30.0%)		1 (1.3%)	13 (16.3%)	

*McNemar test

### PD-1 and PD-L1 expression in primary tumors and matched distant metastases

**Fig. 1:**
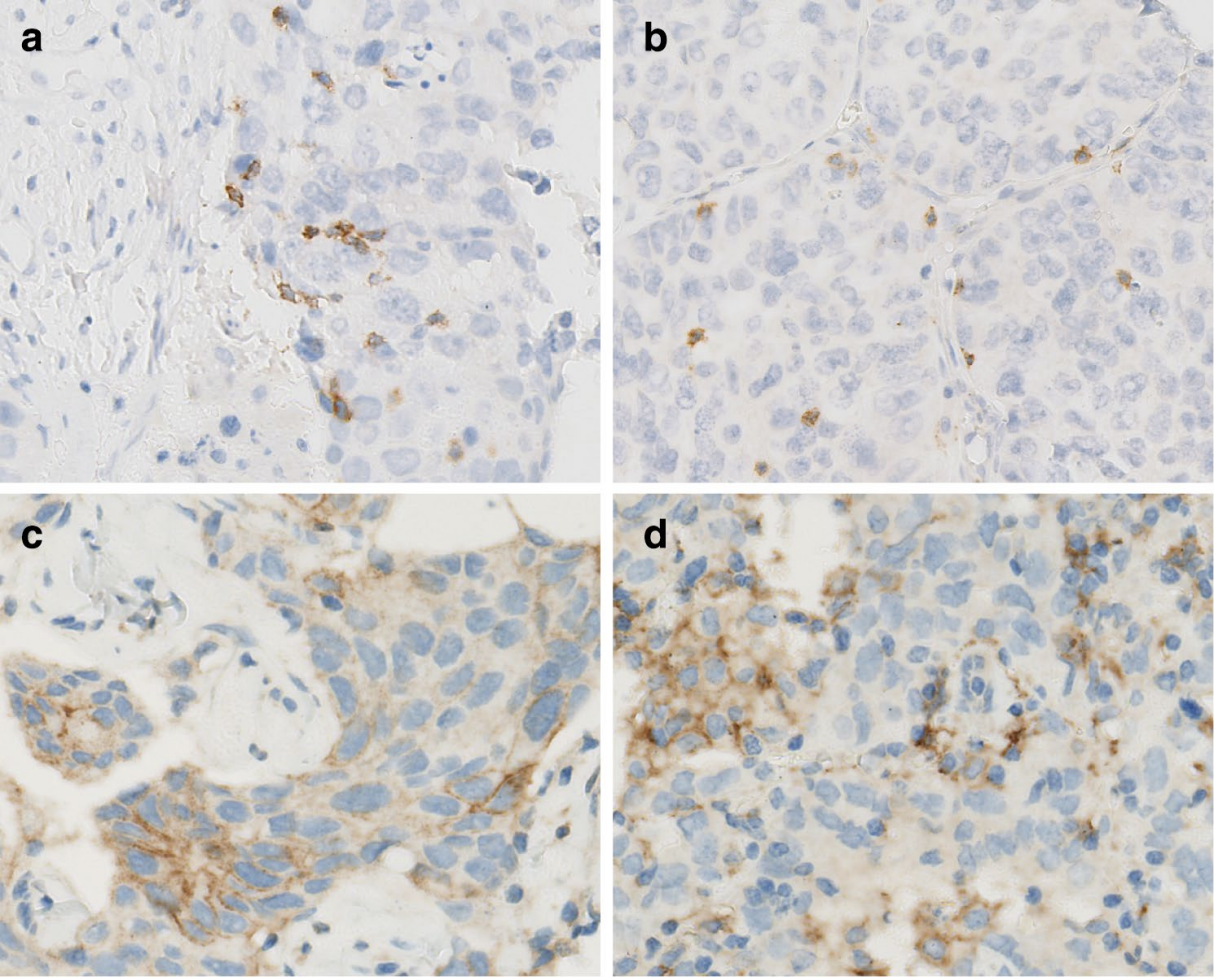
Immunohistochemical positive staining for PD-1 (on immune cells) and PD-L1 (membranous staining on tumor and immune cells) in primary breast tumors and distant metastases. **a** PD-1 in a primary breast tumor. **b** PD-1 in a lung metastasis. **c** PD-L1 in a primary tumor. **d** PD-L1 in a lung metastasis

Figure 1 illustrates positive PD-1 and PD-L1 staining in primary tumors and matched distant metastases. The frequency of PD-1 immune cell expression, and PD-L1 tumor and immune cell expression in primary tumors and metastases is shown in Table 4, and did not differ between both groups.

**Table 4:** PD-1 and PD-L1 expression in primary tumors and non-matched distant metastases

Characteristics	Primary breast tumor	Metastasis	p-value*
PD-1 [no. (%)]			
Negative	46 (43.8)	46 (52.9)	0.211
Positive	59 (56.2)	41 (47.1)	
PD-L1 tumor cells [no. (%)]			
Negative	57 (76.0)	57 (82.6)	0.329
Positive	18 (24.0)	12 (17.4)	
PD-L1 immune cells [no. (%)]			
Negative	43 (57.3)	38 (55.1)	0.785
Positive	32 (42.7)	31 (44.9)	

*Pearson Chi square test

The comparison between expression of PD-1 and PD-L1 between primary tumors and their matched distant metastases is shown in Table 5. For PD-1, 41/82 (50%) primary tumors and metastases were concordant, while 17/82 (20.7%) tumors converted from negative to positive and 24/82 (29.3%) converted from positive to negative (p = 0.349). For tumor PD-L1, 35/49 (71.5%) primary tumors and metastases were concordant, while 3/49 (6.1%) tumors converted from negative to positive and 11/49 (22.4%) converted from positive to negative (p = 0.057). Concerning immune PD-L1, 29/49 (59.2%) primary tumors and metastases were concordant, while 10/49 (20.4%) tumors converted from negative to positive and 10/49 (20.4%) converted from positive to negative (p = 1.000).

**Table 5:** PD-1 and PD-L1 expression in primary breast tumors compared to their matched distant metastases

Distant metastases									
Primary tumor	PD-1			Tumor PD-L1			Immune PD-L1		
	Negative	Positive	p-value*	Negative	Positive	p-value*	Negative	Positive	p-value*
Negative	18 (22.0%)	17 (20.7%)	0.349	31 (63.3%)	3 (6.1%)	0.057	15 (30.6%)	10 (20.4%)	1.000
Positive	24 (29.3%)	23 (28.0%)		11 (22.4%)	4 (8.2%)		10 (20.4%)	14 (28.6%)	

*McNemar test

As shown in Table 6, if PD-1 or PD-L1 (tumor or immune) was inconsistent in the primary breast tumor and metastasis, this was not always the case for the other protein. In only 47.9% of patients, both PD-1 and tumor PD-L1 expression were comparable in the primary tumor and its metastasis, whereas this was the case for 60.9% of patients for both PD-1 and immune PD-L1 expression.

**Table 6:** Receptor conversion of primary breast tumors (P) compared to their matched distant metastases (M)

	PD-1 P = M	PD-1 P ≠ M	p-value*		PD-1 P = M	PD-1 P ≠ M	p-value*
Tumor PD-L1 P = M	17 (37.0%)	16 (34.8%)	0.539	Immune PD-L1 P = M	17 (37.0%)	10 (21.7%)	0.162
Tumor PD-L1 P ≠ M	8 (17.4%)	5 (10.9%)		Immune PD-L1 P ≠ M	8 (17.4%)	11 (23.9%)	

*Pearson Chi square test

### PD-1 and PD-L1 expression for various anatomic sites

When comparing PD-1 and PD-L1 expression between various anatomic sites, least expression for PD-1 was seen in bone and gynecological tract metastases and most frequent PD-1 expression was observed in lung/pleural metastases. For tumor PD-L1 no clear differences were seen, while for immune PD-L1 all three bone metastases did not show any expression, and most expression was observed in lung/pleural metastases, and also both gastrointestinal metastases were positive (Table 7).

**Table 7:** PD-1 and PD-L1 expression in breast cancer metastases at different anatomical sites

Location metastasis	PD-1		Tumor PD-L1		Immune PD-L1	
	Negative no. (%)	Positive No. (%)	Negative no. (%)	Positive no. (%)	Negative no. (%)	Positive no. (%)
Bone (marrow)	4 (100)	0 (0.0)	3 (100)	0 (0.0)	3 (100)	0 (0.0)
Brain	17 (56.7)	13 (43.3)	24 (88.9)	3 (11.1)	16 (59.3)	11 (40.7)
Gastrointestinal^a^	1 (33.3)	2 (66.7)	1 (50.0)	1 (50.0)	0 (0.0)	2 (100)
Gynecological	4 (80.0)	1 (20.0)	3 (100)	0 (0.0)	2 (66.7)	1 (33.3)
Lung/pleural	5 (31.3)	11 (68.8)	8 (61.5)	5 (38.5)	3 (23.1)	10 (76.9)
Skin/subcutis	13 (52.0)	12 (48.0)	13 (81.3)	3 (18.8)	10 (62.5)	6 (37.5)
p-value*	*0.120*		*0.238*		***0.039***	

Bold indicates *p* < 0.05

The italic means that p-value does not belong to the metastasis location

*Pearson Chi square test

^a^For the gastrointestinal subgroup, also liver metastases were included (n = 2)

### Associations between PD-1 and PD-L1 expression and clinicopathological variables

In both primary breast tumors and distant metastases, we did not observe correlations between PD-1 or tumor PD-L1 and hormone receptor status. Immune PD-L1 expression was associated with ER negativity in primary tumors, but not in metastases (Table 8). Further, in primary tumors, there was a correlation between PD-1 and a higher tumor grade (p < 0.001), tumor PD-L1 and triple negative breast cancer (p = 0.003), and a tendency to an association was observed for immune PD-L1 and grade 3 tumors (p = 0.078).

**Table 8:** Association of PD-1 and PD-L1 with selected clinicopathological variables in primary breast tumors and their matched distant metastases

	PD-1		P-value*	Tumor PD-L1		P-value*	Immune PD-L1		P-value*
	Negative no. (%)	Positive no. (%)		Negative no. (%)	Positive no. (%)		Negative no. (%)	Positive no. (%)	
Primary tumor									
ER									
Negative	14 (37.8)	23 (62.2)	0.361	47 (72.3)	18 (27.7)	0.101	29 (44.6)	36 (55.4)	**0.017**
Positive	22 (47.8)	24 (52.2)		62 (83.8)	12 (16.2)		48 (64.9)	26 (35.1)	
PR									
Negative	15 (34.1)	29 (65.9)	0.091	60 (74.1)	21 (25.9)	0.170	43 (53.1)	38 (46.9)	0.639
Positive	20 (52.6)	18 (47.4)		47 (83.9)	9 (16.1)		32 (57.1)	24 (42.9)	
HER2									
Negative	28 (42.4)	38 (57.6)	0.765	83 (76.1)	26 (23.9)	0.129	59 (54.1)	50 (45.9)	0.532
Positive	7 (46.7)	8 (53.3)		25 (89.3)	3 (10.7)		17 (60.7)	11 (39.3)	
Metastasis									
ER									
Negative	17 (43.6)	22 (56.4)	0.126	24 (77.4)	7 (22.6)	0.447	14 (45.2)	17 (54..8)	0.216
Positive	26 (60.5)	17 (39.5)		28 (84.8)	5 (15.2)		20 (60.6)	13 (39.4)	
PR									
Negative	26 (51.0)	25 (49.0)	0.620	35 (81.4)	8 (18.6)	1.000	24 (55.8)	19 (44.2)	0.424
Positive	17 (56.7)	13 (43.3)		16 (80.0)	4 (20.0)		9 (45.0)	11 (55.0)	
HER2									
Negative	34 (52.3)	31 (47.7)	0.963	43 (82.7)	9 (17.3)	0.682	27 (51.9)	25 (48.1)	0.688
Positive	9 (52.9)	8 (47.1)		9 (75.0)	3 (25.0)		7 (58.3)	5 (41.7)	

Bold indicates *p* < 0.05

*Pearson Chi square test

### Survival analysis

**Fig. 2:**
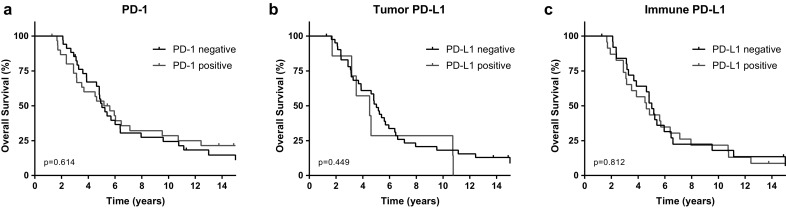
Kaplan–Meier curves for Overall Survival (OS) for PD-1 (**a**), PD-L1 on tumor cells (**b**), and PD-L1 on immune cells (**c**) expression in distant metastases

Sufficient overall survival (OS) data were available for 66 patients. Of those, PD-1 was available for 65 patients, and PD-L1 expression was known for 49 patients. The median follow-up time was 5.1 years (range 1.3–25.9 years). No prognostic values were observed for PD-1 expression (HR 1.150, CI 0.667–1.983, p = 0.614), PD-L1 tumor cell expression (HR 0.730, CI 0.321–1.657, p = 0.449), and PD-L1 immune cell expression (HR 0.930, CI 0.510–1.695, p = 0.812) in distant metastases [Fig. 2]. Also in multivariate analysis, PD-1 and PD-L1 in metastases did not show prognostic values for OS.

In subgroup analysis, we did not see significant differences in overall survival for PD-1 or tumor PD-L1 expression “conversion” (positive to negative, or negative to positive) versus “similarity” (positive to positive, or negative to negative) between primary breast tumor and metastasis (p = 0.848 and p = 0.449). For immune PD-L1, a significantly better OS was observed for patients with PD-L1 negative primary breast tumors that developed PD-L1 positive distant metastases (p = 0.044). The hazard ratio for this group compared to the other three groups combined was 3.013 (CI 1.201–7.561, p = 0.019).

## Discussion

The aim of our study was to compare expression levels of PD-1 on immune cells, and PD-L1 on tumor cells and immune cells, in primary tumors and their matched distant metastases in a large group of breast cancer patients, and to evaluate prognostic values.

We observed that PD-1 and PD-L1 tumor and immune expression were concordant in the distant metastasis and its primary breast tumor in about half to two-third of the patients. In the other part of the patients, PD-1 or PD-L1 negative tumors had developed PD-1 or PD-L1 positive metastases or vice versa. Cimino-Mathews et al. also observed such discordance, but only in two patients and solely for the PD-L1 positive primary tumors [23]. Other studies in breast cancer observed concordance in expression of PD-L1 for primary breast tumors and matched (nodal) metastases in 94% and 100% [22, 29], which might be due to the relatively small size of their cohorts (15 and 17 matched cases, respectively). No previous studies that have been executed, describe PD-1 expression in breast cancer compared to matched distant metastases. Schneider et al. observed high concordance of PD-1 expression in primary head and neck squamous cell carcinoma and matched lymph node metastases (90%), while a slightly lower concordance percentage was found for PD-L1 (70%) [30]. Additionally, we observed that if PD-1 or PD-L1 was discordant in the distant metastasis compared to the primary tumor, this was not necessarily the case for the other protein. As far as we know we are the first to observe this inconsistency. The underlying biology for this discrepancy is yet unknown.

Possible explanations for discrepancies between primary tumors and their matched metastases may be genomic evolution during tumor progression [31], or clonal selection during metastatic process [18]. Otherwise adjuvant (chemo)therapy might influence the tumor microenvironment in breast cancer, and within that process, PD-1 or PD-L1 expression might change. This has been observed before by Yoon et al. where PD-L1 expression levels changed in 4/14 patients treated with neoadjuvant anthracycline- and/or taxane-based chemotherapy [32]. Additionally, Pelekanou et al. compared both TILs and PD-L1 expression in tumors of 58 patients pre- and post-neoadjuvant chemotherapy, and observed an increase of TILs, but a decrease of PD-L1 expression in the post-neoadjuvant therapy tumors [33]. According to these results it may be interesting for future studies to look into more detail into the role of adjuvant therapy in relation to PD-1 and PD-L1 expression conversion in distant metastases.

As we compared PD-1 and PD-L1 expression in different distant anatomic sites, we observed some expression differences between those anatomic sites, in particular for PD-1 and immune PD-L1, although numbers were too small for proper statistical analysis. For example, gynaecological metastases less frequently showed PD-1 expression, while all four bone metastases were negative for PD-1. Previous studies, both in breast cancer as in other cancer types, evaluated PD-L1 expression in lymph node or clustered distant metastases [21, 22, 30, 34], which impedes comparison of our observations. However, Ogiya et al. studied PD-L1 expression in brain metastases only, and observed comparable results with regard to PD-L1 expression frequency as we did (45% vs. 41%) [24].

Furthermore, in subgroup analysis, we found a correlation between PD-1 positivity and higher tumor grade in primary breast tumors, which is in line with literature [8, 14, 16]. We did not observe other associations between PD-1 expression and selected clinicopathological variables, both in primary breast tumors as in metastases. Concerning PD-L1 in primary breast tumors, we observed associations of immune PD-L1 with ER negativity and a tendency of a correlation with grade 3 tumors, and for tumor PD-L1 an association with triple negative breast cancer. These findings are in line with the literature, since PD-L1 positivity is often associated with worse clinicopathological characteristics like larger tumor size, higher tumor grade and ER and PR negativity [7, 9–11, 13, 15]. The relatively small size of our cohort for subgroup analyses might explain we only observed these associations in primary tumors and not in metastases.

In survival analysis we did not observe significant differences concerning OS for PD-1 and PD-L1 on both tumor and immune cells. As far as we know, no other study has assessed prognostic value of PD-1 and PD-L1 expression in metastases. In primary breast tumors, the prognostic value of PD-1 and PD-L1 is equivocal, since previous studies described different outcomes for both PD-1 [14, 16], as PD-L1 [6, 7, 10, 11, 15]. Furthermore, a significantly better OS for patients with PD-L1 negative tumors that developed PD-L1 positive metastases was observed in the present study. However, subgroups were small, so we do not want to speculate on the biological background of this observation. Further evaluation in bigger groups could be interesting.

Comparison of PD-1 and PD-L1 expression between different studies remains difficult due to use of different antibodies, scoring methods and use of different cut-off values. For example, cut-off values varying from 1 to 50% were used, leading to different percentages of positive tumors [17]. Additionally, PD-L1 is expressed on both tumor cells and immune cells. Whereas most studies evaluate PD-L1 on tumor cells, recent studies also considered PD-L1 expression on immune cells [10, 17], like we did. Therefore, further standardization of (scoring) methods for the use of PD-1 and PD-L1 seems to be important.

Since PD-1 and PD-L1 expression are thought to be heterogeneous, it might be a limitation that we used TMAs to evaluate expression instead of full tissue slides. We therefore used three cores per tissue block that represented different tumor areas. Additionally, we verified the use of TMAs by comparing these PD-L1 scores of ten patients with PD-L1 scores on whole slides. In half of the patients complete agreement was noticed, while the other half only showed minimal differences. The strength of our study lays in the relatively large group of primary tumors and matched distant metastases of breast cancer patients.

To conclude, PD-1 and PD-L1 (on tumor and immune cells) positivity is concordant between primary tumors and distant metastases in only half to two-third of the breast cancer patients. In the other part of the patients PD-1 or PD-L1 negative tumors developed PD-1 or PD-L1 positive metastases or vice versa. Furthermore, patients with immune PD-L1 negative breast tumors that develop PD-L1 positive distant metastases seem to have the best overall survival. This illustrates the need of reassessing PD-1 and tumor and immune PD-L1 expression in biopsies of distant metastases to optimize the usefulness of these biomarkers.

## Abbreviations

PD-1: Programmed death-1
PD-L1: Programmed death-ligand 1
TILs: Tumor infiltrating lymphocytes
